# Resting Energy Expenditure in Patients with Extreme Obesity: Comparison of the Harris–Benedict Equation with Indirect Calorimetry

**DOI:** 10.3390/jcm13195993

**Published:** 2024-10-08

**Authors:** Anna Jílková, Barbora Lampová, Ondřej Kádě, Lenka Kouřimská, Diana Chrpová, Iveta Kaiserová, Martin Matoulek

**Affiliations:** 1 Department of Microbiology, Nutrition and Dietetics, Faculty of Agrobiology, Food and Natural Resources, Czech University of Life Sciences Prague, 165 21 Prague, Czech Republic; lampova@af.czu.cz (B.L.); kourimska@af.czu.cz (L.K.); chrpovad@af.czu.cz (D.C.); 2 3rd Internal Department of Endocrinology and Metabolism, General University Hospital, 1st Faculty of Medicine, Charles University, 128 08 Prague, Czech Republic; ondrej.kade@lf1.cuni.cz (O.K.); iveta.kaiserova@lf1.cuni.cz (I.K.); martin.matoulek@lf1.cuni.cz (M.M.)

**Keywords:** obesity, energy expenditure, weight loss, indirect calorimetry

## Abstract

**Background**: The main objective of the work was the analysis and description of data on body composition and resting energy expenditure (REE) values of selected groups of patients with obesity whose REE measurement results using indirect calorimetry reached a level below 95% of the predicted REE calculated using the Harris–Benedict (H–B) equation. The sub-goals were to describe the dependence of body composition on the size of the REE and to find out if the deviations between the number of the total measured REE and the REE calculated using H–B in the adapted group (patients with altered REE values, lower than expected caused by long caloric restriction) are significant. **Methods**: For the research, 71 (39 women and 32 men) patients treated in obesitology were selected. Patients underwent the measurement of resting metabolism using indirect calorimetry (IC) and body composition measurement on the bioimpedance device and, at the same time, the value of resting metabolism was calculated for everyone using the H–B equation. The whole group was divided into five groups according to the deviation of the measurement using IC and the calculation of the H–B equation. **Results**: In the total set of examined individuals, there were 32.4% with a reduced REE value compared to the REE calculation according to the H–B equation, which corresponds to 23 individuals. In the adapted group, the average measured REE was 2242 ± 616 kcal compared to the H–B calculation of 2638 ± 713 kcal. Statistically, these results were not significant, but a high case-to-case variation was found. The highest deviation from the H–B predictive calculation was −42% and +43% in the whole research group. The amount of muscle tissue in the adapted group averaged 44.3 ± 11.9 kg and the amount of fat-free mass (FFM) 77.9 ± 20.1 kg. When statistically testing the dependence of REE on FFM and muscle tissue in the adapted group, a strong correlation was found. **Conclusions**: The H–B equation alone is not suitable for setting a suitable diet therapy for an individual with obesity. In order to select and characterize a group of adapted individuals, it will be necessary to use other methods or a larger research sample, and preferably examine and divide patients with specific comorbidities or include their health status.

## 1. Introduction

Obesity is one of the most widespread multifactorial diseases on our planet today. Up to several hundred million new overweight or obese individuals are added annually, and currently more than two and a half billion of the world’s population is affected, with a suggested four billion until 2035 [1,2]. Obesity stands as one of the most prevalent multifactorial diseases, arising from the interplay of numerous obesogenic factors in an individual’s environment. While some cases of obesity stem from genetic predispositions or underlying medical conditions, the majority are attributed to poor lifestyle choices and insufficient physical activity. Obesity not only poses health risks to individuals, including an increased likelihood of developing comorbidities and elevated mortality rates, but also presents a significant socioeconomic burden. Achieving weight reduction necessitates attaining a negative energy balance, achieved through either reduced food intake or increased physical activity. However, obesity treatment is a complex matter, and in many cases achieving or maintaining weight loss proves challenging. In addition to conventional approaches such as dietary interventions and exercise, other methods like psychotherapy, pharmacotherapy, and bariatric surgery, which continue to show growing potential, may be beneficial [3,4].

Obesity is a multifactorially influenced disease and knowledge of resting energy expenditure contributes to understanding the factors of its development [5]. Resting energy expenditure, often referred to as basal metabolic rate (BMR), is the largest component of total energy expenditure (TEE), accounts for the energy expended in a fasting state, resting in a lying position at a neutral ambient temperature, free of physical and psychological stress and to maintain vital physiological functions such as respiration, circulation, and cellular metabolism, as measured in healthy objects [6,7,8]. REE is taken under less strict conditions than BMR and it is approximately 10% higher; the difference takes into account the shorter fasting period and thus the oxidation of energy substrates during oral intake [9,10]. The accurate assessment of REE in patients with obesity not only provides insights into their metabolic status but also plays a crucial role in designing personalized weight management interventions and therapeutic strategies [7,11].

The relationship between obesity and REE has been a subject of intense investigation due to its implications for energy balance regulation and weight management. While it is well established that obesity is associated with alterations in energy metabolism, the precise nature and extent of these changes remain a topic of ongoing research. Understanding the factors influencing REE in individuals with obesity, including body composition, hormonal regulation, and genetic predisposition, is essential for unraveling the complexities of obesity-associated metabolic dysregulation [12,13]. In this study, these patients are called “adapted”, which comes from the phenomenon of metabolic adaptation, when an individual is exposed to long-term caloric restriction, which leads to a reduction of the individual’s basal metabolism below the expected values and, depending on this, a reduced ability to reduce weight (mainly fat component) [14,15].

Moreover, accurate assessment methods are imperative for evaluating REE in patients with obesity. Traditional predictive equations, such as the Harris–Benedict equation, may lack accuracy when applied to overweight individuals due to differences in body composition and metabolic activity [16,17]. Advanced techniques such as indirect calorimetry offer a more precise means of measuring REE by directly assessing oxygen consumption and carbon dioxide production. However, the practicality and accessibility of these methods in clinical settings warrant consideration [7,18]. Indirect calorimetry is also used to detect underestimation and overestimation of the patient’s intake and to set appropriate therapy [8,19,20].

Although there are studies that compare predictive equations with the results of indirect calorimetry, there is still a lack of data for the assessment of individuals with extreme obesity or examined individuals originating from the Czech Republic. In this study, we wanted to analyze and evaluate the data of patients with obesity measured by indirect calorimetry and bioimpedance equipment. The primary objective was to analyse and describe data on body composition and resting energy expenditure (REE) values among selected groups of individuals with obesity. Specifically, the study focuses on individuals whose REE measurements using indirect calorimetry fell below 95% of the predicted REE calculated using the Harris–Benedict equation. In this way, we could better identify and characterize individuals with a tendency toward metabolic adaptation to low energy intake and set up appropriate therapy [21]. The REE value measured using indirect calorimetry is considered the reference method [22,23]. Co-objectives were to determine the relationship between body composition in adapted patients and the measured REE value and compare the REE measurement results obtained through indirect calorimetry and with those calculated using the Harris–Benedict equation among different groups.

## 2. Materials and Methods

### 2.1. Design

The research was conducted at the 3rd Internal Department of Endocrinology and Metabolism, General University Hospital, 1st Faculty of Medicine, Charles University in Prague. The data collection took place from December 2021 to June 2023, with a total of 71 individuals undergoing indirect calorimetry measurements and requiring selection criteria. These patients were monitored and had regular consultations with an obesitologist and nutritional therapist, and this measurement was part of their treatment. The selection criteria for research subjects were BMI (Body mass Index) ≥ 30 kg/m^2^, successful completion of bioimpedance body composition testing conducted simultaneously, and successful completion of indirect calorimetry testing (steady state achieved). A Cortex MetaLyzer 3B (CORTEX Biophysik GmbH, Leipzig, Germany, 2020) device was used for indirect calorimetry measurements, and an InBody 230 (Biospace Co., Seoul, Republic of Korea, 2012) device was used for tetrapolar bioimpedance analysis. These results were compared with the resting metabolic rate calculation using the H–B equation (10% of the calculated basal metabolic rate was added for metabolic rate calculation) [24].

In line with the set goals, all patients with obesity were measured and only then the group with measured REE below 95% of the expected value (the adapted group) was selected. Additionally, the entire dataset was divided into groups based on the degree of deviation from the predictive H–B equation and compared the characteristics of each group. The REE value measured by indirect calorimetry was considered the reference method.

### 2.2. Participants

The research sample consisted of patients from the Department of Internal Medicine III, Department of Endocrinology and Metabolism, First Faculty of Medicine, Charles University, and General University Hospital in Prague. Patients with obesity with a BMI ≥ 30 kg/m^2^ who were either hospitalized or attending consultations with nutrition therapists were selected for inclusion in the study. A total of 71 individuals met the criteria and were included in the research, comprising 39 women (55%) and 32 men (45%).

### 2.3. Body Composition and Indirect Calorimetry Measurement

Conditions for undergoing indirect calorimetry and bioimpedance measurements:

- Refrain from eating for 12 h before the measurements, with the last meal being light;
- Drink only water after the last meal, avoiding alcohol, coffee, black and green tea;
- Avoid smoking or using any nicotine products before the measurements;
- Avoid strenuous physical activity 24 h before the measurements;
- Arrive well in advance and sit quietly for at least 10 min (physical and mental rest affect measurement quality);
- Avoid medications that may affect the measurements (e.g., beta-blockers, antidepressants, anti-obesity drugs, and others). If possible, take these medications after the measurements;
- The patient should remain completely still during the 20–30-min measurement period but should not fall asleep;
- Ensure an optimal temperature around 23 °C, ventilate if the temperature is higher, and minimize airflow during the measurements;
- Contraindications include pregnancy and the presence of a defibrillator (for bioimpedance);
- Women should not be measured several days before and during menstruation;
- All devices and objects in direct contact with the patient are disinfected after each measurement;
- Patients are informed about all conditions well in advance and briefed on the measurement procedure by healthcare personnel and sign the informed consent.

Both conducted measurements were successive, taking place in the morning from 7 a.m. to 9 a.m., allowing patients to better adhere to the established conditions. The InBody device measurement preceded the indirect calorimetry measurement. Patients were measured barefoot in their underwear. For indirect calorimetry, the device was calibrated before the first measurement of the day. A mask with a disposable turbine of appropriate size was fitted to the patient’s face. This was followed by a 20-min measurement in a lying position, during which a five-minute interval of steady state was detected by the device (if all measurement conditions were met). The device then analysed oxygen consumption and carbon dioxide production and calculated the patient’s REE using the Weir equation [18,20,25].

### 2.4. Data Analysis

The obtained data were exported from the databases of the devices and statistically analyzed. The studied individuals were divided into 5 groups based on the deviation of the measured REE from the calculated REE according to the Harris–Benedict equation. Subsequently, the results were evaluated using the program STATISTICA 12 (StatSoft CR s.r.o., 2017, Tulsa, OK, USA). The level of significance was defined as *p* < 0.05. Tests for descriptive statistics were used to characterize the groups and the basic distribution of participants. All descriptive data were described using the mean ± standard deviation (SD). The statistical analysis of the data involved the application of one-way analysis of variance (ANOVA), followed by post-hoc analyses using Scheffe’s method for finding differences between groups and *t*-tests. The correlation test was used to compare the relationship between the degree of adaptation and body composition components.

## 3. Results

### 3.1. Achievement of the Main Objective

**Figure 1:**
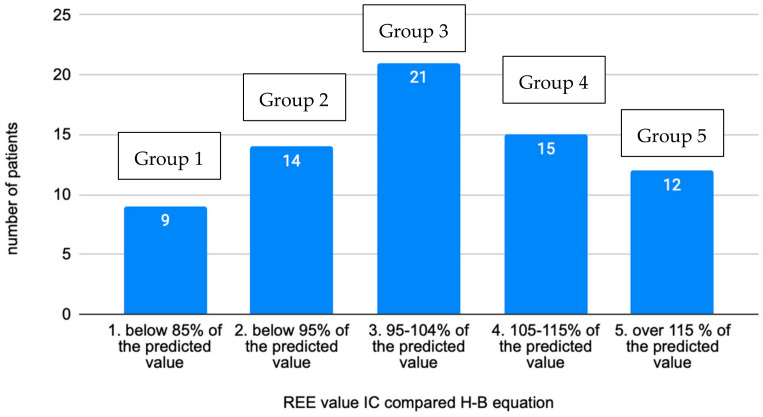
Distribution of individuals into groups based on the measured REE value compared to calculation by the H–B equation (*n* = 71).

The research dataset was divided into five groups based on the level of the measured REE value compared to the calculated REE according to the Harris–Benedict equation (Figure 1). These groups were defined based on REE results as follows:

- Below 85% of predicted value (group 1).
- 85–94% of predicted value (group 2).
- 95–104% of predicted value (group 3).
- 105–115% of predicted value (group 4).
- Above 115% of predicted value (group 5).

**Figure 2:**
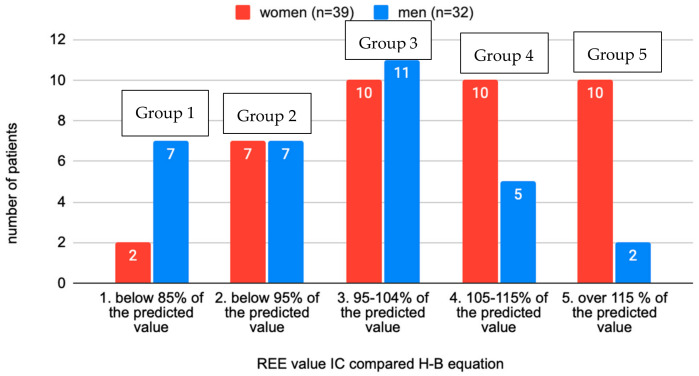
Distribution of individuals according to the REE rate measured and calculated by the H–B equation between sexes (*n* = 71).

In the total sample of examined individuals, 32.4% had a reduced REE compared to the calculation according to the Harris–Benedict equation, which corresponds to 23 individuals. The distribution of groups appears to be normal or Gaussian. The highest deviations from the predictive H–B calculation was −42% and +43%. Most patients fell into group 3, which would agree with a deviation of ±10% for the calculation according to H–B. Regarding the distribution of the sexes in the groups, an interesting observation was the increasing number of women in higher-numbered groups and, conversely, the decreasing number of men (Figure 2).

The average BMI in the sample was 45.2 ± 11.6 kg/m^2^, with women exhibiting a higher average BMI (46.9 kg/m^2^) than men (43.2 kg/m^2^). The lowest measured BMI was 30 kg/m^2^, while the highest was 85.6 kg/m^2^. Patients were also categorized according to the degree of obesity into groups: obesity grade I (30.0–34.9 kg/m^2^), with 19.7% of individuals; obesity grade II (35.0–39.9 kg/m^2^), with 25.4% of individuals; and the majority falling into obesity grade III (>40.0 kg/m^2^), comprising 54.9% of individuals. The average age of the patients was 53.3 ± 13.9 years, with the youngest participant being 20 years old and the oldest being 79 years old. The average age was lower for women (52.2 years) compared to men (54.7 years). Regarding body weight, the average value for the entire sample was 137.0 ± 37.5 kg, with men having a higher average weight (144.0 ± 36.2 kg) than women (131.2 ± 38.0 kg). The lightest individual weighed 71.0 kg, while the heaviest weighed 235.7 kg. Further details are presented in Table 1.

Table 2 and Table 3 describe descriptive characteristics of women and men. Statistically significant differences (*p* < 0.05) analysed by *t*-tests were found between the sexes in the parameters REE level compared to H–B calculation (*p* = 0.0078), deviations from norm (*p* = 0.0051) and quality (*p* = 0.0350).

Characteristics of individual groups did not differ significantly according to statistical analysis of the data involved the application of one-way analysis of variance (ANOVA), followed by post-hoc analysis using Scheffe’s method and *t*-tests. Group 2 had the lowest average age (50.3 ± 16.6 years), while group 4 had the highest (55.5 ± 10.3 years). Regarding the average height in the groups, group 5 had the lowest values (165.7 ± 14.8 cm) and group 1 had the highest values (180.3 ± 8.8 cm). The average weight was also lowest in group 1 (121.7 ± 28.9 kg) and highest in group 3 (149.6 ± 35.2 kg).

Among other characteristics describing body composition were muscle mass, fat, water, fat-free mass (FFM), and percentage of fat. These values also did not provide a certain characterization of the group. For example, the overall lowest muscle mass value was in group 5 (36.2 ± 9.1 kg), but mainly due to the lower average height, the highest muscle mass was measured in group 1 (49.5 ± 10.7 kg). The highest average fat amount was in group 3, at 67.4 ± 23.8 kg, and the lowest in group 1, at 54.0 ± 33.4 kg. These characteristics, as well as other results describing individual groups, are listed in Table 4. Although the REE values calculated using the REE H–B and REE measured did differ, these differences were statistically significant only in group 5 (*p =* 0.0073). In groups 1 to 4, the differences were not statistically significant, likely due to the large variances of values and therefore also high standard deviations (group 1: *p* = 0.0527, group 2: *p =* 0.3563, group 3: *p =* 0.9872, group 4: *p =* 0.2513).

The following part of this study focused mainly on groups 1 and 2, with reduced REE values compared to the calculation of the predictive equation. We describe and try to identify these groups of patients mainly for the purpose of solving the problem of obesity and its complications. These patients are very often unsuccessful in terms of weight reduction and their treatment is more demanding, often fails, and involves the collaboration of specialists from different fields [26]. These groups did not exhibit different characteristics compared to the other groups (Table 5). The individual parameters comparing every group together are added to Supplementary Material: Tables with *p*-values of the compared groups. REE values of groups 1 and 2 together calculated using the REE H–B and REE measured did not differ significantly (*p* = 0.0501). When comparing data from groups 1 and 2 together to the rest groups *p*-value was significant in REE measured (kcal/d) in groups 1 + 2 compared to 3 (*p* = 0.026912) and 5 (*p* = 0.018629), in groups 1 + 2 compared 5 for IB-muscle (kg) (*p* = 0.047133), IB-FFM (kg) (*p* = 0.048327), REE (kcal/d) (*p* = 0.037902).

### 3.2. Correlation of Body Composition and REE

To determine the relationship between body composition and REE, a correlation test was chosen with a significance level of alpha < 0.05. Correlation values indicate the strength of the relationship and range from −1 to 1. For adapted patients in groups 1 and 2, a strong correlation was found between the amount of muscle mass, FFM, water, and the size of the measured REE (*p* < 0.05). Conversely, the relationship between fat percentage and REE size was not statistically significant (*p* = 0.2700). This means that for adapted individuals, the more muscle mass they have, the higher the REE will be. The sizes of the compared variables are listed in Table 6.

## 4. Discussion

Our study confirmed the existence of a large group of people with altered energy expenditure, which can lead to incorrect treatment settings. However, the difference between the predictive equation and indirect calorimetry results showed statistical significance only in group 5, not in the others. This could be due to the large variance of values within the group, varying individually from person to person. It is likely that with a larger sample size, these results (especially in groups 1 and 2) would become statistically significant, as the *p*-value was close to 0.05 even for these groups. For a significant number of individuals, the predictive equations are not entirely accurate and may not be the best tool for calculating resting energy expenditure. Each individual’s energy expenditure is influenced differently, with factors like body composition playing a significant role. For instance, basal metabolic rate (BMR) accounts for 50–80% of total energy expenditure, with body composition and fat-free mass (FFM) playing pivotal roles [12,26]. Predictive equations used to calculate BMR often overlook variations in body composition, leading to considerable inaccuracies, especially among individuals with abnormal body compositions like obesity or athletes [11]. Consequently, indirect calorimetry, which accurately measures REE through the analysis of respiratory gases, is increasingly being regarded as the gold standard. For instance, in a clinical study examining 1440 hospitalized individuals, the REE value calculated using the Harris–Benedict equation and other predictive equations deviated by ±430–570 kcal compared to the value measured using indirect calorimetry [27]. Another study investigated the comparison of several types of predictive equations with indirect calorimetry but in patients with cancer. This study found an underestimation of REE by the Harris–Benedict equation of 27% compared to IC which is perhaps even more troubling with this type of diagnosis [28]. However, many studies still consider the H–B equation to be one of the most accurate for patients with obesity [29,30,31,32].

Reduced basal (resting) metabolism in individuals with obesity may result from repeated attempts at weight reduction. Prolonged low energy intake relative to BMR leads to metabolic adaptation, wherein BMR decreases below the predicted value. In this study, 32.4% of individuals with obesity were found to be adapted in this manner. Study of Poli et al. (2016) compared resting metabolism measured using indirect calorimetry with REE values calculated using predictive equations in a sample of women with obesity. They found that 27% of individuals exhibited metabolic adaptation [33]. This phenomenon of metabolic adaptation is primarily attributed to alterations in hormone concentrations involved in regulating body composition, such as thyroid hormones, leptin, testosterone, and insulin, whose concentrations decrease, as well as cortisol and ghrelin, whose concentrations increase. These hormonal changes can persist even after successful weight reduction attempts [34,35].

The distribution of the sexes in groups according to the deviation from indirect calorimetry showed an increasing number of women in the group with increased REE and the opposite in men. This could be attributed to the better physical condition of the women in the research sample or the higher adaptation of men to low energy intake and thus lower REE values. There were no similar studies found that supported this trend.

The magnitude of REE is heavily influenced by an individual’s body composition. Both individuals with high FFM and those with large amounts of adipose tissue exhibit high REE, as it is dependent on total body weight. That means the heavier the weight, the higher the REE. But comparing two same weight individuals, REE would be higher in individual with higher FFM. However, most predictive equations, such as the Harris–Benedict equation, do not account for individual body composition, resulting in varying levels of accuracy [36,37]. The review by Madden et al. (2016) does not recommend evaluating individuals with obesity with predictive equations, the Mifflin equation appears to be the only suitable one, which also evaluates body composition, but even so its inaccuracy is 10–25% [17]. Our study evaluated the association between muscle mass, FFM, and resting metabolism in a group of adapted patients, finding that higher muscle and fat-free mass correlated with increased resting energy expenditure. Hirsch et al. (2017) reached similar conclusions in a study examining the correlation between body composition, hormone levels, and resting metabolism in 49 individuals, considering differences between men and women and the composition of various body segments [35]. These findings underscore the importance of physical activity in maintaining and increasing muscle mass in adapted patients, where dietary restrictions may play a minor role and could potentially exacerbate the loss of active muscle mass [7,38].

Discrepancies comparing deviations between measured REE using indirect calorimetry and calculation using the Harris–Benedict equation could be attributed to either adaptation to low energy expenditure or increased muscle mass in the group with elevated REE values. It is also possible that these deviations were influenced by non-compliance with pre-measurement instructions, patient non-co-operation, and the inability to achieve a state of mental and physical calmness during measurement [39]. Additionally, the health status of the individuals was not taken into account, leading to the inclusion of individuals with various comorbidities [40]. Another limitation of the study could be the classification of patients based solely on BMI, which does not account for body fat percentage, and the overall lack of attention to sex differences—individual parameters, such as resting metabolism, can vary significantly between genders. To examine both sexes separately, a larger sample size would be required, and this expansion and more analyses are planned for future studies. We were unable to statistically compare the differences between the sexes due to the sample size and because of parameters that can naturally show different values for the sexes. The higher quality of testing among women may be explained by better preparation, adherence to the set conditions, and greater co-operation from female participants. The higher measured REE in women could also be attributed to their better physical condition.

In order to select and characterize a group of adapted individuals, it will be necessary to use other methods or a larger sample of individuals, and at the same time preferably examine patients without comorbidities or include their health status. It would be interesting to compare these results with other methods (such as DXA—Dual-energy X-ray absorptiometry) or results using other devices or different predictive equations [41]. Nevertheless, despite the high demands on personnel and strict examination protocols, indirect calorimetry can accurately determine an individual’s resting energy expenditure and aid in the development of an appropriate weight reduction plan.

## 5. Conclusions

The results calculated from the Harris–Benedict equation and the measured values may, in some cases, differ significantly. Therefore, it is not advisable to always rely on these equations, and using other, more reliable methods for determining REE in subsequent obesity therapy is recommended. For this group of adapted individuals with obesity, the main therapy will be to increase the amount of FFM through regular physical activity.

## Figures and Tables

**Table 1:** Descriptive characteristics of the sample (*n* = 71) (measured parameters, mean ± standard deviation, minimum–maximum).

Parameter	Mean	Minimum	Maximum
Age (years)	53.3 ± 13.9	20.0	79.0
Height (cm)	174.0 ± 12.3	128.0	205.0
Weight (kg)	137.0 ± 37.5	71.0	235.7
BMI (kg/m^2^)	45.2 ± 11.7	30.0	85.5
BSA (m^2^)	68.5 ± 9.9	46.0	92.8
REE H–B (kcal/d/h)	103.6 ± 26.5	65.0	177.0
REE H–B (kcal/d)	2486 ± 635	1560	4248
REE measured (kcal/d)	2509 ± 629	1369	4010
REE H–B–REE measured (kcal)	−23.5 ± 408	−1010	1418
REE level compared to H–B calculation (%)	102.2 ± 16.3	57.8	143.2
REE/Body Weight (kcal/d/kg)	18.7 ± 3.3	10.4	27.4
REE/BSA (kcal/d/m^2^)	1027 ± 173	601	1526
Deviations from Norm (%)	2.4 ± 16.3	−42.0	43.0
Quality (%)	66.3 ± 22.1	13.0	100.0
RQ	0.8 ± 0.1	0.7	1.1
BF (min)	14.6 ± 4.1	4.0	24.0
IB-muscle (kg)	42.7 ± 11.2	20.4	77.6
IB-fat (kg)	61.6 ± 25.4	8.1	124.8
IB-water (kg)	55.5 ± 13.8	27.8	97.9
IB-FFM (kg)	75.2 ± 18.7	37.8	133.5
WHR	1.0 ± 0.2	0.3	1.3
Fat Amount (%)	44.0 ± 9.5	6.8	57.0

Abbreviations: BMI—body mass index, REE—resting energy expenditure, IB—InBody, FFM—fat free mass, WHR—waist hip ratio, H–B—Harris–Benedict equation, kcal—calorie, d—day, BF—breathing frequency, RQ—respiration quotient, BSA—body surface area.

**Table 2:** Descriptive characteristics of the women (*n* = 39) (measured parameters, mean ± standard deviation, minimum–maximum).

Parameter	Mean	Minimum	Maximum
Age (years)	52.2 ± 14.7	20.0	79.0
Height (cm)	166.9 ± 9.5	128.0	182.0
Weight (kg)	131.2 ± 38.1	71.0	221.0
BMI (kg/m^2^)	46.9 ± 12.6	31.9	85.5
BSA (m^2^)	65.2 ± 9.4	46.0	84.4
REE H–B (kcal/d/h)	91.2 ± 18.8	65.0	134.0
REE H–B (kcal/d)	2188 ± 452	1560	3216
REE measured (kcal/d)	2326 ± 537	1369	3535
REE H–B–REE measured (kcal)	−138.5 ± 345.6	−922	789
REE level compared to H–B calculation (%)	106.8 ± 16.3	64.6	143.2
REE/Body Weight (kcal/d/kg)	18.3 ± 3.6	10.4	27.4
REE/BSA (kcal/d/m^2^)	1003 ± 169	601	1414
Deviations from Norm (%)	7.2 ± 16.0	−35.0	43.0
Quality (%)	71.3 ± 21.6	13.0	100.0
RQ	0.8 ± 0.1	0.7	1.0
BF (min)	15.3 ± 4.1	8.0	24.0
IB-muscle (kg)	37.1 ± 8.9	20.4	59.9
IB-fat (kg)	64.9 ± 24.5	32,9	124.8
IB-water (kg)	48.6 ± 11.1	27.8	77.5
IB-FFM (kg)	65.8 ± 14.6	37.8	102.6
WHR	1.0 ± 0.2	0.4	1.3
Fat Amount (%)	48.6 ± 5.4	34.7	57.0

Abbreviations: BMI—body mass index, REE—resting energy expenditure, IB—InBody, FFM—fat free mass, WHR—waist hip ratio, H–B—Harris–Benedict equation, kcal—calorie, d—day, BF—breathing frequency, RQ—respiration quotient, BSA—body surface area.

**Table 3:** Descriptive characteristics of the men (*n* = 32) (measured parameters, mean ± standard deviation, minimum–maximum).

Parameter	Mean	Minimum	Maximum
Age (years)	54.7 ± 13.0	23.0	73.0
Height (cm)	182.5 ± 9.7	168.0	205.0
Weight (kg)	144.0 ± 36.2	92.0	235.7
BMI (kg/m^2^)	43.2 ± 10.2	30.0	72.7
BSA (m^2^)	72.4 ± 9.1	58.5	92.7
REE H–B (kcal/d/h)	118.7 ± 26.8	79.0	177.0
REE H–B (kcal/d)	2848 ± 643	1896	4248
REE measured (kcal/d)	2731 ± 669	1942	4010
REE H–B–REE measured (kcal)	116 ± 440	−1010	1418
REE level compared to H–B calculation (%)	96.6 ± 14.7	57.8	133.7
REE/Body Weight (kcal/d/kg)	19.2 ± 2.9	11.8	26.2
REE/BSA (kcal/d/m^2^)	1055 ± 177	730	1526
Deviations from Norm (%)	−3.5 ± 14.8	−42.0	34.0
Quality (%)	60.2 ± 21.4	23.0	96.0
RQ	0.9 ± 0.1	0.8	1.1
BF (min)	13.6 ± 4.0	4.0	22.0
IB-muscle (kg)	49.6 ± 9.9	36.6	77.6
IB-fat (kg)	57.6 ± 26.3	8.1	120.8
IB-water (kg)	64.0 ± 12.1	48.5	97.9
IB-FFM (kg)	86.8 ± 16.5	65.4	133.5
WHR	1.0 ± 0.2	0.3	1.2
Fat Amount (%)	38.4 ± 10.4	6.8	52.6

Abbreviations: BMI—body mass index, REE—resting energy expenditure, IB—InBody, FFM—fat free mass, WHR—waist hip ratio, H–B—Harris–Benedict equation, kcal—calorie, d—day, BF—breathing frequency, RQ—respiration quotient, BSA—body surface area.

**Table 4:** Descriptive statistics comparing individual groups (measured parameters, mean ± standard deviation, *n* = 71).

Parameter	Group 1	Group 2	Group 3	Group 4	Group 5
Age (years)	53.3 ± 14.0	50.3 ± 16.6	53.9 ± 11.9	55.5 ± 10.3	52.9 ± 18.6
Height (cm)	180.3 ± 8.8	174.8 ± 14.1	177.1 ± 11.8	171.5 ± 7.1	165.7 ± 14.8
Weight (kg)	142.4 ± 39.6	137.6 ± 42.9	149.6 ± 35.2	127.6 ± 38.2	121.7 ± 28.9
BMI (kg/m^2^)	43.9 ± 12.9	44.6 ± 11.7	47.8 ± 10.7	43.9 ± 15.3	44.1 ± 7.7
REE H–B (kcal/d)	2741 ± 698	2571 ± 740	2679 ± 645	2250 ± 474	2150 ± 433
REE measured (kcal/d)	2116 ± 564	2323 ± 655	2676 ± 637	2468 ± 545	2781 ± 599
IB-muscle (kg)	49.5 ± 10.7	40.9 ± 11.8	46.7 ± 11.7	40.1 ± 8.2	36.2 ± 9.1
IB-fat (kg)	54.0 ± 33.4	65.5 ± 26.7	67.4 ± 23.8	57.6 ± 27.5	57.5 ± 17.3
IB-water (kg)	64.1 ± 13.4	53.1 ± 14.6	60.2 ± 14.4	52.4 ± 10.1	47.5 ± 11.6
IB-FFM (kg)	87.0 ± 18.0	72.1 ± 19.7	81.7 ± 19.5	70.9 ± 13.1	64.3 ± 15.6
WHR	0.9 ± 0.3	1.0 ± 0.2	1.0 ± 0.1	1.0 ± 0.2	1.0 ± 0.1
Fat amount (%)	36.3 ± 14.7	46.8 ± 6.8	44.4 ± 9.6	43.1 ± 8.3	46.8 ± 6.3

Abbreviations: BMI—body mass index, REE—resting energy expenditure, IB—InBody, FFM—fat free mass, WHR—waist hip ratio, H–B—Harris–Benedict equation, kcal—calorie, d—day. There were no statistically significant differences between numbers on the same row in any group (*p* ≤ 0.05).

**Table 5:** Descriptive statistics for both groups 1 and 2 together (measured parameters, mean ± standard deviation, *n* = 23).

Parameter	Mean	Minimum	Maximum
Age (years)	51.5 ± 15.4	20.0	69.0
Height (cm)	177.0 ± 12.4	155.0	205.0
Weight (kg)	139.5 ± 40.8	81.1	235.7
BMI (kg/m^2^)	44.3 ± 11.9	32.3	74.7
BSA (m^2^)	69.9 ± 10.5	50.8	92.8
REE H–B (kcal/d)	2638 ± 713	1560	4248
REE measured (kcal/d)	2242 ± 617	1369	3686
REE H–B–REE measured (kcal)	396 ± 291	92.0	1418
REE level compared to H–B calculation (%)	85.4 ± 8.7	57.8	94.2
IB-muscle (kg)	44.3 ± 11.9	24.3	67.4
IB-fat (kg)	61.0 ± 29.3	8.1	120.8
IB-water (kg)	57.4 ± 14.9	32.2	86.7
IB-FFM (kg)	77.9 ± 20.1	43.9	114.9
WHR	1.0 ± 0.2	0.3	1.2
Fat amount (%)	42.7 ± 11.5	6.8	57.0

Abbreviations: BMI—body mass index, REE—resting energy expenditure, IB—InBody, FFM—fat free mass, WHR—waist hip ratio, H–B—Harris–Benedict equation, kcal—calorie, d—day.

**Table 6:** Correlation of REE with body composition variables in groups 1 and 2 (correlation (group 1 + 2, *n* = 23, *p* < 0.05)).

Variable × REE Measured (kcal/d)	Correlation Value (r)
IB-muscle (kg)	0.7452 (*p* = 0.0000)
IB-water (kg)	0.7381 (*p* = 0.0000)
IB-FFM (kg)	0.7306 (*p* = 0.0000)
Fat amount (%)	0.2398 (*p* = 0.2700)

Abbreviations: REE—resting energy expenditure, IB—InBody, FFM—fat free mass, H–B—Harris–Benedict equation, kcal—calorie, d—day.

## Data Availability

The original contributions presented in the study are included in the article/supplementary material, further inquiries can be directed to the corresponding author/s.

## Supplementary Materials

The following supporting information can be downloaded at: https://www.mdpi.com/article/10.3390/jcm13195993/s1, File S1: Tables with *p*-values of the compared groups.
