# Supplementary material for: Resting Energy Expenditure in Patients with Extreme Obesity: Comparison of the Harris–Benedict Equation with Indirect Calorimetry
Source: J Clin Med. 2024 Oct 8;13(19):5993. doi: 10.3390/jcm13195993 (PMC11478319; doi:10.3390/jcm13195993)
Supplement: Supplementary file 1 [file jcm-13-05993-s001.zip › Supplementary File S1.pdf]

Response 2: Thank you for this point. I will try to describe as I can, I apologize for mistakes and misunderstanding in the response. I will be very glad for any recommendations and more advice in case of improving the answers.

We focused primarily on groups 1 and 2, where I reported statistically significant differences between these groups (1+2) and the others. As additional material, I have created tables with individual values of the investigated parameters and p-values, comparing these parameters across groups. Comments follow below the individual tables, and statistically significant differences or results approaching a p-value of 0.05 are mentioned.

Parameter	Comparison: Group 1 (n=9) vs. Group 2 (n=14), mean±standard deviation, p-value				
	Mean (1)	Mean (2)	p - value	Standard deviation (1)	Standard deviation (2)
Quality (%)	70,9	67,8	0,72	22,6	18,4
IB-muscle (kg)	49,5	40,9	0,09	10,7	11,8
IB-fat (kg)	54,0	65,5	0,37	33,4	26,7
IB-water (kg)	64,1	53,1	0,08	13,4	14,6
IB-FFM (kg)	87,0	72,1	0,08	18,0	19,7
Fat amount (%)	36,3	46,8	0,03	14,7	6,8
Age (years)	54,3	51,1	0,63	14,0	16,7
Height (cm)	180,6	174,8	0,29	8,8	14,1
Weight (kg)	142,4	137,6	0,79	39,6	42,9
BMI (kg/m²)	43,9	44,6	0,91	12,9	11,7
REE H-B (kcal/d)	2741	2571	0,59	698	740
REE measured (kcal/d)	2115	2323	0,44	563	655

Abbreviations: BMI – body mass index, REE – resting energy expenditure, IB – InBody, FFM – fat free mass, WHR – waist hip ratio, H-B – Harris Benedict equation, kcal – calorie, d – day.

The statistical significance between the group 1 and 2 was found in parameter of the fat amount (p=0.03). The amount of water (p=0.08), FFM (p=0.08), and muscle mass (p=0.09), in kilograms almost approached the p-value of 0.05. With a larger sample size, it is possible that these values would become less significant due to large individual variations.

Parameter	Comparison: Group 1 (n=9) vs. Group 3 (n=21), mean±standard deviation, p-value				
	Mean (1)	Mean (3)	p - value	Standard deviation. (1)	Standard deviation (3)
Quality (%)	70,9	67,0	0,67	22,6	22,2
IB-muscle (kg)	49,5	46,7	0,54	10,7	11,7
IB-fat (kg)	54,0	67,4	0,22	33,4	23,8
IB-water (kg)	64,1	60,2	0,50	13,4	14,4
IB-FFM (kg)	87,0	81,7	0,49	18,0	19,5
Fat amount (%)	36,3	44,4	0,08	14,7	9,6

Age (years)	54,3	54,7	0,94	14,0	12,1
Height (cm)	180,6	177,1	0,44	8,8	11,8
Weight (kg)	142,4	149,6	0,62	39,6	35,2
BMI (kg/m²)	43,9	47,8	0,41	12,9	10,7
REE H-B (kcal/d)	2741	2678	0,81	698	645
REE measured (kcal/d)	2115	2675	0,03	563	637

The statistical significance between the group 1 and 3 was found in parameter of the REE measured (kcal/d) (p=0.03). The amount of fat (p=0.08) also approached the p-value near 0.05. This can be explained by higher amount of FFM, muscle mass and total weight in group 3.

Parameter	Comparison: Group 1 (n=9) vs. Group 4 (n=15), mean±standard deviation, p-value				
	Mean (1)	Mean (4)	p - value	Standard deviation (1)	Standard deviation (4)
Quality (%)	70,9	67,7	0,76	22,6	25,2
IB-muscle (kg)	49,5	40,1	0,02	10,7	8,2
IB-fat (kg)	54,0	57,6	0,78	33,4	27,5
IB-water (kg)	64,1	52,4	0,02	13,4	10,1
IB-FFM (kg)	87,0	70,9	0,02	18,0	13,1
Fat amount (%)	36,3	43,1	0,15	14,7	8,3
Age (years)	54,3	56,3	0,69	14,0	10,4
Height (cm)	180,6	171,5	0,01	8,8	7,1
Weight (kg)	142,4	127,6	0,38	39,6	38,2
BMI (kg/m²)	43,9	43,9	0,99	12,9	15,3
REE H-B (kcal/d)	2741	2249	0,05	698	473
REE measured (kcal/d)	2115	2467	0,14	563	544

The statistical significance between the group 1 and 4 was found in parameter of the fat amount (p=0.03). The amount of water (p=0.02), FFM (p=0.02), and muscle mass (p=0.02) and height (p=0.01). REE H-B was 0.05 and that is due to the REE counted by equation depends on parameters of height, weight and age. Higher height, higher weight and lower age mean higher REE values. This reason is the same in next cases of the statistical significancy connected to the REE H-B.

Parameter	Comparison: Group 4 (n=15) vs. Group 5 (n=12), mean±standard deviation, p-value				
	Mean (4)	Mean (5)	p - value	Standard deviation (4)	Standard deviation (5)
Quality (%)	67,7	58,0	0,31	25,2	22,8
IB-muscle (kg)	40,1	36,2	0,25	8,2	9,1
IB-fat (kg)	57,6	57,5	0,99	27,5	17,3

IB-water (kg)	52,4	47,5	0,25	10,1	11,6
IB-FFM (kg)	70,9	64,3	0,24	13,1	15,6
Fat amount (%)	43,1	46,8	0,21	8,3	6,3
Age (years)	56,3	53,8	0,65	10,4	18,5
Height (cm)	171,5	165,7	0,19	7,1	14,8
Weight (kg)	127,6	121,7	0,66	38,2	28,9
BMI (kg/m²)	43,9	44,1	0,97	15,3	7,7
REE H-B (kcal/d)	2249	2150	0,58	473	432
REE measured (kcal/d)	2467	2780	0,17	544	599

The statistical significance between the group 4 and 5 was not found maybe due to similarity of these groups.

Parameter	Comparison: Group 1+2 (n=23) vs. Group 5 (n=12), mean±standard deviation, p-value				
	Mean (1+2)	Mean (5)	p - value	Standard deviation (1+2)	Standard deviation (5)
Quality (%)	69,0	58,0	0,15	19,7	22,8
IB-muscle (kg)	44,3	36,2	0,05	11,9	9,1
IB-fat (kg)	61,0	57,5	0,70	29,3	17,3
IB-water (kg)	57,4	47,5	0,05	14,9	11,6
IB-FFM (kg)	77,9	64,3	0,05	20,1	15,6
Fat amount (%)	42,7	46,8	0,25	11,5	6,3
Age (years)	52,3	53,8	0,81	15,5	18,5
Height (cm)	177,0	165,7	0,02	12,4	14,8
Weight (kg)	139,5	121,7	0,19	40,8	28,9
BMI (kg/m²)	44,3	44,1	0,95	11,9	7,7
REE H-B (kcal/d)	2637	2150	0,04	712	432
REE measured (kcal/d)	2242	2780	0,02	616	599

The statistical significance between the group 1 + 2 and other was described in the results section.

Parameter	Comparison: Group 1 (n=9) vs. Group 5 (n=12), mean±standard deviation, p-value				
	Mean (1)	Mean (5)	p - value	Standard deviation (1)	Standard deviation (5)
Quality (%)	70,9	58,0	0,21	22,6	22,8
IB-muscle (kg)	49,5	36,2	0,01	10,7	9,1
IB-fat (kg)	54,0	57,5	0,76	33,4	17,3
IB-water (kg)	64,1	47,5	0,01	13,4	11,6
IB-FFM (kg)	87,0	64,3	0,01	18,0	15,6

Fat amount (%)	36,3	46,8	0,04	14,7	6,3
Age (years)	54,3	53,8	0,94	14,0	18,5
Height (cm)	180,6	165,7	0,01	8,8	14,8
Weight (kg)	142,4	121,7	0,18	39,6	28,9
BMI (kg/m²)	43,9	44,1	0,98	12,9	7,7
REE H-B (kcal/d)	2741	2150	0,03	698	432
REE measured (kcal/d)	2115	2780	0,02	563	599

The statistical significance between the group 1 and 5 was found in parameter of the fat amount (p=0.04), amount of water (p=0.01), FFM (p=0.01), muscle mass (p=0.01), height (p=0.01), REE H-B (p=0.03) and REE measured (p=0.02). This was due to the higher values of REE of the group 5 and the lowest of group 1 with slowed metabolism.

Parameter	Comparison: Group 2 (n=14) vs. Group 3 (n=21), mean±standard deviation, p-value				
	Mean (2)	Mean (3)	p - value	Standard deviation (2)	Standard deviation (3)
Quality (%)	67,8	67,0	0,91	18,4	22,2
IB-muscle (kg)	40,9	46,7	0,16	11,8	11,7
IB-fat (kg)	65,5	67,4	0,83	26,7	23,8
IB-water (kg)	53,1	60,2	0,16	14,6	14,4
IB-FFM (kg)	72,1	81,7	0,16	19,7	19,5
Fat amount (%)	46,8	44,4	0,44	6,8	9,6
Age (years)	51,1	54,7	0,46	16,7	12,1
Height (cm)	174,8	177,1	0,60	14,1	11,8
Weight (kg)	137,6	149,6	0,37	42,9	35,2
BMI (kg/m²)	44,6	47,8	0,41	11,7	10,7
REE H-B (kcal/d)	2571	2678	0,65	740	645
REE measured (kcal/d)	2323	2675	0,12	655	637

The statistical significance between the group 2 and 3 was not found maybe due to similarity of these groups.

Parameter	Comparison: Group 2 (n=14) vs. Group 4 (n=15), mean±standard deviation, p-value				
	Mean (2)	Mean (4)	p - value	Standard deviation (2)	Standard deviation (4)
Quality (%)	67,8	67,7	0,99	18,4	25,2
IB-muscle (kg)	40,9	40,1	0,82	11,8	8,2
IB-fat (kg)	65,5	57,6	0,44	26,7	27,5
IB-water (kg)	53,1	52,4	0,88	14,6	10,1
IB-FFM (kg)	72,1	70,9	0,85	19,7	13,1

Fat amount (%)	46,8	43,1	0,21	6,8	8,3
Age (years)	51,1	56,3	0,31	16,7	10,4
Height (cm)	174,8	171,5	0,43	14,1	7,1
Weight (kg)	137,6	127,6	0,51	42,9	38,2
BMI (kg/m²)	44,6	43,9	0,89	11,7	15,3
REE H-B (kcal/d)	2571	2249	0,17	740	473
REE measured (kcal/d)	2323	2467	0,52	655	544

The statistical significance between the group 2 and 3 was not found maybe due to similarity of these groups.

Parameter	Comparison: Group 1+2 (n=23) vs. Group 3 (n=21), mean±standard deviation, p-value				
	Mean (1+2)	Mean (3)	p - value	Standard deviation (1+2)	Standard deviation (3)
Quality (%)	69,0	67,0	0,75	19,7	22,2
IB-muscle (kg)	44,3	46,7	0,50	11,9	11,7
IB-fat (kg)	61,0	67,4	0,43	29,3	23,8
IB-water (kg)	57,4	60,2	0,52	14,9	14,4
IB-FFM (kg)	77,9	81,7	0,53	20,1	19,5
Fat amount (%)	42,7	44,4	0,58	11,5	9,6
Age (years)	52,3	54,7	0,58	15,5	12,1
Height (cm)	177,0	177,1	0,99	12,4	11,8
Weight (kg)	139,5	149,6	0,38	40,8	35,2
BMI (kg/m²)	44,3	47,8	0,32	11,9	10,7
REE H-B (kcal/d)	2637	2678	0,84	712	645
REE measured (kcal/d)	2242	2675	0,03	616	637

The statistical significance between the group 1 + 2 and other was described in the results section.

Parameter	Comparison: Group 2 (n=14) vs. Group 5 (n=12), mean±standard deviation, p-value				
	Mean (2)	Mean (5)	p - value	Standard deviation (2)	Standard deviation (5)
Quality (%)	67,8	58,0	0,24	18,4	22,8
IB-muscle (kg)	40,9	36,2	0,27	11,8	9,1
IB-fat (kg)	65,5	57,5	0,38	26,7	17,3
IB-water (kg)	53,1	47,5	0,29	14,6	11,6
IB-FFM (kg)	72,1	64,3	0,28	19,7	15,6

Fat amount (%)	46,8	46,8	0,98	6,8	6,3
Age (years)	51,1	53,8	0,70	16,7	18,5
Height (cm)	174,8	165,7	0,12	14,1	14,8
Weight (kg)	137,6	121,7	0,29	42,9	28,9
BMI (kg/m^2)	44,6	44,1	0,90	11,7	7,7
REE H-B (kcal/d)	2571	2150	0,10	740	432
REE measured (kcal/d)	2323	2780	0,08	655	599

The statistical significance between the group 2 and 5 on REE measured (p=0.08) almost approached 0.05. Again, it is due to the highest value of REE measured in the group 5.

Parameter	Comparison: Group 3 (n=21) vs. Group 4 (n=15), mean±standard deviation, p-value				
	Mean (3)	Mean (4)	p - value	Standard deviation (3)	Standard deviation (4)
Quality (%)	67,0	67,7	0,93	22,2	25,2
IB-muscle (kg)	46,7	40,1	0,07	11,7	8,2
IB-fat (kg)	67,4	57,6	0,26	23,8	27,5
IB-water (kg)	60,2	52,4	0,08	14,4	10,1
IB-FFM (kg)	81,7	70,9	0,07	19,5	13,1
Fat amount (%)	44,4	43,1	0,67	9,6	8,3
Age (years)	54,7	56,3	0,68	12,1	10,4
Height (cm)	177,1	171,5	0,11	11,8	7,1
Weight (kg)	149,6	127,6	0,08	35,2	38,2
BMI (kg/m^2)	47,8	43,9	0,37	10,7	15,3
REE H-B (kcal/d)	2678	2249	0,04	645	473
REE measured (kcal/d)	2675	2467	0,31	637,3	544

The statistical significance between the group 3 and 4 was found in parameter of the REE H-B (p=0.04). The amount of water (p=0.08), FFM (p=0.07), and muscle mass (p=0.07) and weight (p=0.08) almost approached the p-value of 0.05. This is again due to the dependency of REE H-B to the weight, age and height.

Parameter	Comparison: Group 3 (n=21) vs. Group 5 (n=12), mean±standard deviation, p-value				
	Mean (3)	Mean (5)	p - value	Standard deviation (3)	Standard deviation (5)
Quality (%)	67,0	58,0	0,28	22,2	22,8
IB-muscle (kg)	46,7	36,2	0,01	11,7	9,1
IB-fat (kg)	67,4	57,5	0,21	23,8	17,3
IB-water (kg)	60,2	47,5	0,01	14,4	11,6
IB-FFM (kg)	81,7	64,3	0,01	19,5	15,6

Fat amount (%)	44,4	46,8	0,45	9,6	6,3
Age (years)	54,7	53,8	0,86	12,1	18,5
Height (cm)	177,1	165,7	0,02	11,8	14,8
Weight (kg)	149,6	121,7	0,03	35,2	28,9
BMI (kg/m^2)	47,8	44,1	0,30	10,7	7,7
REE H-B (kcal/d)	2678	2150	0,02	645	432
REE measured (kcal/d)	2675	2780	0,65	637	599

The statistical significance between the group 3 and 5 were found in parameters of the IB-muscle (kg) (p=0.01), the amount of water (p=0.01), FFM (p=0.01), height (p=0.02), weight (p=0.03) and REE measured (p=0.02). With a larger sample size, it is possible that these values would become less significant due to large individual variations. The REE counted by equation depends on parameters of height, weight and age. Higher height, higher weight and lower age mean higher REE values.

Parameter	Comparison: Group 1+2 (n=23) vs. Group 4 (n=15), mean±standard deviation, p-value				
	Mean (1+2)	Mean (4)	p - value	Standard deviation (1+2)	Standard deviation (4)
Quality (%)	69,0	67,7	0,86	19,7	25,2
IB-muscle (kg)	44,3	40,1	0,24	11,9	8,2
IB-fat (kg)	61,0	57,6	0,72	29,3	27,5
IB-water (kg)	57,4	52,4	0,26	14,9	10,1
IB-FFM (kg)	77,9	70,9	0,24	20,1	13,1
Fat amount (%)	42,7	43,1	0,89	11,5	8,3
Age (years)	52,3	56,3	0,39	15,5	10,4
Height (cm)	177,0	171,5	0,13	12,4	7,1
Weight (kg)	139,5	127,6	0,38	40,8	38,2
BMI (kg/m^2)	44,3	43,9	0,92	11,9	15,3
REE H-B (kcal/d)	2637	2249	0,07	712	473
REE measured (kcal/d)	2242	2467	0,26	616	544

Statistical significance between the group 1 + 2 and other was described in the results section.
